# Automated separation of overlapping fingermarks by non-negative matrix factorization of DESI mass spectrometry imaging data

**DOI:** 10.1007/s00216-026-06574-3

**Published:** 2026-05-28

**Authors:** Palle Villesen, Kirstine Lykke Nielsen, Kim Frisch

**Affiliations:** 1 Department of Clinical Medicine, Aarhus University, Palle Juul-Jensens Boulevard 82, 8200 Aarhus N, Denmark; 2 Section for Bioinformatics and Computational Biology (BiRC), Aarhus University, Universitetsbyen 81, Building 1872, 8000 Aarhus C, Denmark; 3 Department of Forensic Medicine, Aarhus University, Palle Juul-Jensens Boulevard 99, 8200 Aarhus N, Denmark

**Keywords:** Non-negative matrix factorization, Mass spectrometry imaging, DESI-MSI, Forensic fingermarks, Overlapping fingermarks, Chemometrics

## Abstract

Overlapping fingermarks present a significant challenge in forensic investigations, as traditional optical methods cannot separate superimposed ridge patterns from multiple donors. We present an automated and unsupervised approach using non-negative matrix factorization (NMF) to separate overlapping fingermarks from desorption electrospray ionization mass spectrometry imaging (DESI-MSI) data by exploiting the distinct chemical signatures of individual donors. DESI-MSI datasets containing 100–200,000 pixels with intensity measurements up to 5000 mass-to-charge channels were decomposed using NMF to identify spatially coherent molecular signatures. Using PCA as a baseline, NMF produced superior results with sharper spatial boundaries between fingermark regions, directly interpretable non-negative molecular signatures, and parts-based decomposition that naturally represents the additive nature of overlapping chemical deposits. The completely unsupervised analysis successfully identified distinct fingermark donors and their associated molecular profiles without requiring prior knowledge of donor number or chemical characteristics. The approach also successfully resolved a simulated four-donor scenario, demonstrating scalability beyond two overlapping fingermarks. We recommend NMF as the preferred method for detailed forensic fingermark analysis, since it offers forensic practitioners an automated, interpretable tool for analyzing complex fingermark evidence where multiple deposits overlap. The approach is likely to extend to other MS imaging areas such as document analysis.

**Graphical abstract:**

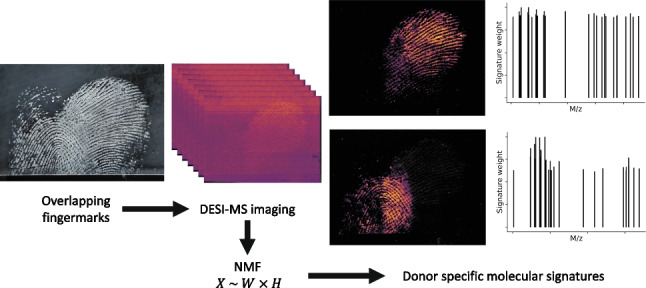

**Supplementary Information:**

The online version contains supplementary material available at 10.1007/s00216-026-06574-3.

## Introduction

Latent fingermarks are among the most valuable forms of physical evidence in forensic investigations. Beyond identification through ridge pattern comparison, fingermarks contain rich chemical information that can reveal details about the depositor, including lifestyle factors, medication use, and environmental exposures [1, 2]. However, critical challenges arise when fingermarks from multiple individuals overlap at a crime scene. Traditional optical examination methods struggle to separate superimposed ridge patterns, and the value of such evidence can be severely compromised. This paper presents a computational approach using non-negative matrix factorization (NMF) to separate overlapping fingermarks by exploiting their distinct chemical signatures determined by mass spectrometry imaging (MSI).

MSI has emerged as a transformative tool for fingermark analysis, capable of simultaneously capturing spatial ridge detail and molecular composition in a single measurement. Desorption electrospray ionization mass spectrometry imaging (DESI-MSI) is particularly well-suited for forensic applications: charged solvent droplets are sprayed onto the sample surface, desorbing molecular species that are then analyzed based on their mass-to-charge (m/z) ratio [3, 4]. The technique requires minimal sample preparation, can be applied to fingermarks on forensic gelatin lifters used routinely at crime scenes, and preserves the spatial information essential for ridge pattern analysis [5]. Each pixel in a DESI-MSI dataset contains a complete mass spectrum, creating a rich, high-dimensional dataset where spatial location and MS signals are intrinsically linked.

The fundamental principle, enabling separation of overlapping fingermarks, is that everyone’s fingermark residue has a characteristic chemical profile. This profile comprises endogenous compounds secreted through eccrine, apocrine, and sebaceous glands, including lipids, fatty acids, amino acids, and peptides, as well as exogenous compounds acquired through environmental contact with personal care products, foods, medications, and other materials [6–8]. Person-specific variation in gland activity, skin chemistry, diet, and lifestyle creates distinctive molecular fingermarks that persist even when ridge patterns overlap. Previous studies have demonstrated that MSI can exploit these chemical differences to visualize individual fingermarks within overlapping deposits, with recent work showing successful separation based on compounds such as personal care product additives unique to individual donors [5, 9].

A DESI-MSI dataset of a fingermark may contain hundreds of thousands of pixels, each with intensity measurements across thousands of m/z values. Extracting meaningful patterns from such high-dimensional data requires sophisticated computational methods. Principal component analysis (PCA) has been widely applied to MSI data and can identify major sources of variance, but its orthogonality constraint and allowance for negative values limit its interpretability for non-negative intensity measurements [10, 11]. Supervised methods such as single compound analysis (e.g., t-test or Anova) require prior knowledge of group labels (i.e., groups of pixels known to be from different donors) and cannot discover molecular mixes or molecular interactions. What is needed is an unsupervised method that respects the non-negative nature of mass spectrometry data and produces interpretable, spatially coherent components with associated molecular signatures.

Non-negative matrix factorization (NMF) offers a principled solution to this analytical challenge. NMF decomposes a data matrix into two non-negative factor matrices: one describing molecular signatures (which compounds contribute to each component) and one describing spatial weights (how strongly each component is expressed at each pixel) [12, 13]. The non-negativity constraint aligns with the physical reality that molecular abundances cannot be negative, and the resulting components represent additive mixtures of molecular sources rather than deviations from a mean. For overlapping fingermarks, this means NMF should be able to identify the distinct chemical signature of each donor and represent overlapping regions as weighted sums of these signatures, directly reflecting the physical process of co-deposition.

Several characteristics of NMF make it particularly attractive for forensic fingermark analysis. First, the parts-based decomposition naturally handles spatially localized, overlapping patterns because components are additive rather than orthogonal [14–16]. Second, the non-negative loadings for each m/z value are directly interpretable: each component can be characterized by the specific molecules that define it, facilitating identification of donor-specific markers or contaminants of forensic interest. Third, NMF handles sparse data gracefully, which is important given that many masses have zero intensity at most pixels due to spatial heterogeneity and detection limits. Fourth, the additive model corresponds to the biological reality that fingermark residue is a mixture of contributions from multiple glands and environmental sources, each present in non-negative proportions that vary across the fingermark surface [6].

Despite these theoretical advantages, NMF has been rarely applied in forensic MSI applications. While the method has been applied to biological tissue imaging for cancer research and metabolic studies [17–20], no published work has systematically evaluated NMF for forensic fingermark analysis with quantitative cross-sample validation. This gap is notable given that the characteristics of fingermark MSI data, overlapping patterns from multiple sources, sparse measurements, the need for interpretable molecular signatures, and non-negative intensity values, align extremely well with NMF’s strengths.

In this work, we apply NMF to DESI-MSI data from latent fingermarks and compare its performance to PCA as a widely used baseline. We evaluate spatial coherence and boundary definition, interpretability of the molecular signatures associated with each component, and the ability to identify distinct chemical environments including separate fingermark donors. We demonstrate that NMF produces spatially coherent components that directly correspond to distinct chemical sources, with each component accompanied by a molecular signature that can be examined for compounds of forensic interest. The completely unsupervised nature of the analysis means that neither the number of donors nor their chemical characteristics need to be specified in advance; NMF discovers the underlying structure without requiring labeled training samples. This approach offers forensic practitioners a fully automated and interpretable tool for analyzing complex fingermark evidence where traditional methods fall short.

## Methods

### Sample collection and DESI-MSI data acquisition

Fingermark data were acquired using desorption electrospray ionization mass spectrometry imaging (DESI-MSI) as described in detail earlier [5]. Briefly, latent fingermarks were deposited on glass microscope slides, enhanced with white magnetic powder, and lifted onto gelatin lifters. DESI-MSI of the gelatin lifters was performed using a Waters Xevo G2-XS QToF mass spectrometer equipped with a DESI-XS ion source, acquiring data in positive ionization mode across a mass range of 50–1200 m/z with 50 µm ✕ 50 µm spatial resolution. The resulting datasets contain approximately 100–200,000 pixels with intensity measurements for up to 5000 mass-to-charge (m/z) channels per pixel. Each slide required approximately 11–15 h to acquire (scan rate 200 µm/s, approximately 1.75 min/mm^2^).

Five pairs of natural overlapping fingermarks from six donors were analyzed (Table 1). Three pairs had been previously published in Frisch et al. [5] and two new pairs were collected for this study. Natural fingermarks were deposited using a random finger “as presented,” i.e., without applying any specific procedure or preparation, such as washing hands and deliberately touching the face prior to deposition.

**Table 1:** Sample details

Slide ID	Pair ID^a^	Acq. date^b^	Aging code^c^	Donor pair^d^	#pixels^e^	#m/z columns^f^
1	Pair 4	2023-02-15	0-0-0	D5 (F)/D6 (M)	170,200	5000
2	Pair 17	2023-10-19	14-14-14	D6 (M)/D5 (F)	182,780	5000
3	Pair 7	2023-11-08	0-0-0	D11 (F)/D12 (F)	187,488	5000
4	Pair 19	2024-02-02	0-0-1	D2 (M)/D6 (M)	124,800	5000
5	Pair 20	2024-02-08	0-0-0	D5 (F)/D1 (F)	176,928	4494

^a^Data for Pairs 4, 17, and 7 are from Frisch et al. [5]. Data for Pairs 19 and 20 are previously unpublished but obtained using the same method as described in Frisch et al. [5]. ^b^Date when DESI-MSI data was acquired. ^c^The aging code X-Y-Z refers to the time a fingermark was left on the original surface until powder treatment and lift (X and Y denote the first and second deposition, respectively), and the time the lifted fingermarks were stored on the gelatin support until analysis (Z); all in days and at indoor conditions. ^d^Donor number refers to Frisch et al. [5]; F = female, M = male. ^e^total number of pixels in the scanned area (using a 50 µm ✕ 50 µm spatial resolution). ^f^Number of ions (m/z) extracted from raw data. The HDI software removes m/z channels below the signal detection threshold (threshold = 10) prior to export. Slide 5 yielded 4494 channels with detectable signal rather than 5000, reflecting lower overall signal abundance on that slide; the acquisition settings were identical across all slides

The samples were acquired on different dates over a period of approximately one year, providing independent replicates for validation of the computational approach.

### Data preprocessing

All data processing was performed in R (version 4.5) using custom analysis pipelines. Raw DESI-MSI data were pre-processed using Waters HDI software (ver. 1.6) as described in Frisch et al. [5]. The processed imaging data were exported as text files containing pixel coordinates (x, y) and intensity values for each m/z channel (default format). Data were imported and stored as three data structures: (1) An intensity matrix (N × M), where each row corresponds to one pixel spectrum (N ≈ 100–200,000 pixels) and each column corresponds to one m/z feature (M ≈ 4494–5000 m/z channels) with the majority of values being 0 (sparse data). (2) A pixel coordinate table (N × 2) with x,y positions for each pixel. (3) An m/z feature table (M × 1) with the m/z value for each column of the intensity matrix. Intensity values were log-transformed using the log1p transformation (log(1 + x)) to reduce the dynamic range and stabilize variance while handling zero values appropriately. The three data structures were stored in qs2 format using the qs2 package for efficient storage and fast read/write operations.

### Non-negative matrix factorization

NMF was performed using the RcppML package [21], which implements a fast alternating least squares algorithm optimized for large-scale sparse data which minimizes the squared Frobenius norm (mean squared error of the original and reconstructed matrix). The data matrix A (N × M, where N ≈ 200,000 pixels and M ≈ 5000 m/z columns) was decomposed into two non-negative factor matrices: W (N × K) containing spatial weights for each pixel, and H (K × M) containing molecular signatures for each NMF component. No L1 regularization was applied (L1 parameters set to 0 for both W and H). The number of components was set to K = 30, convergence tolerance to 10^–6^, and maximum iterations to 500. Random seed was fixed at 0 for reproducibility. High-precision fitting of NMF required up to 25 min (HPC node, 4 cores, 100 GB RAM; 500 iterations, convergence tolerance 1 × 10^−6^). NMF signatures were rescaled so that the maximum coefficient within each signature equaled 1, and weights were similarly rescaled so that the maximum weight for each component equaled 1. This rescaling facilitates comparison across components while preserving relative relationships within each signature and weight distribution.

### Principal component analysis

For comparative analysis, PCA was performed using the irlba package. PCA was performed both with centering (standard PCA) and without centering to evaluate the effect of mean removal on spatial component structure. The uncentered variant maintains non-negative values and enables direct comparison with NMF, which also operates on non-negative log-transformed intensities. Data were not scaled in either case. The top K = 30 principal components were extracted, matching the number of NMF components for direct comparison. Pixel scores were computed by projecting the original data matrix onto the rotation matrix (loadings), producing spatial maps of component expression comparable to NMF weight matrices.

### Component ordering by Moran’s I

PCA components are naturally ordered by variance explained, where NMF components are randomly ordered. To make spatial plots more comparable, we ordered NMF and PCA components by decreasing Moran’s I spatial autocorrelation statistic [22, 23] using the moran() function from the spdep package. Moran’s I ranges from − 1 (dispersed pattern) through 0 (random) to + 1 (strongly clustered), with higher values indicating spatially coherent patterns. Differences in Moran’s I distributions between methods were assessed using two-sided non-parametric Wilcoxon rank-sum tests.

### Visualization and analysis

Spatial maps were generated using ggplot2 with raster-based plotting for efficiency with large pixel counts. For visualization, each component’s spatial weights (NMF) or scores (PCA) were scaled to the maximum absolute value across all pixels, ensuring that components with small weights are visible in the plots alongside high-weight components. NMF component weights were visualized using the inferno colormap from the viridis package, which provides perceptually uniform color gradients suitable for representing non-negative intensity values on a dark background. PCA scores were visualized using a diverging color scale (mako-black-inferno) centered at zero to distinguish positive and negative loadings, reflecting the bidirectional nature of PCA components. Component correlation was assessed using Spearman correlation of weight vectors (for spatial co-localization). Hierarchical clustering of correlation matrices was performed using Ward’s method with correlation distance, visualized as heatmaps using the pheatmap package. For each NMF component, the top contributing molecules were identified by ranking the signature coefficients, providing interpretable molecular profiles associated with each spatial pattern.

### Software and reproducibility

All analyses were implemented as R Markdown documents enabling reproducible execution of the complete workflow from raw data to final figures. Key packages included tidyverse for data manipulation, qs2 for efficient data storage, RcppML for NMF, irlba for PCA, spdep for Moran’s I, pheatmap for heatmap visualization, and cowplot for figure composition. Random seeds were explicitly set for all stochastic operations to ensure reproducibility. All data and code are available at Zenodo (see data availability section).

### Simulated four-fingermark dataset

To evaluate whether NMF can separate more than two overlapping fingermarks, we constructed a simulated four-fingermark dataset by combining two existing acquisition sessions. Slide 1 (donors D5/D6, acquired 2023-02-15) was flipped vertically and merged with slide 3 (donors D11/D12, acquired 2023-11-08), yielding a dataset containing fingermarks from four different donors. These two slides were selected for their similar pixel dimensions and aspect ratios. The merging procedure comprised the following steps: (1) both preprocessed datasets were cropped to their maximum common pixel dimensions; (2) m/z values were binned to 0.1 Da to align mass channels across acquisition sessions, with within-slide duplicate channels summed in raw (non-log) intensity scale; (3) a shared global normalization factor was computed from the top 10% strongest pixels and applied to both slides to make their overall signal levels comparable while preserving spatial contrast; (4) the union of m/z bins across both slides was taken, assigning zero intensity where a bin was absent in one slide; and (5) the two aligned matrices were combined by pixel-wise sum in raw intensity scale, followed by re-application of the log1p transformation. NMF was then applied to the merged dataset with K = 40 components (increased from K = 30 for individual slides to accommodate the additional donors), using the same parameters as described above (tolerance 10−6, 500 iterations, seed = 0, no L1 regularization). 

## Results

### Data overview

**Fig. 1:**
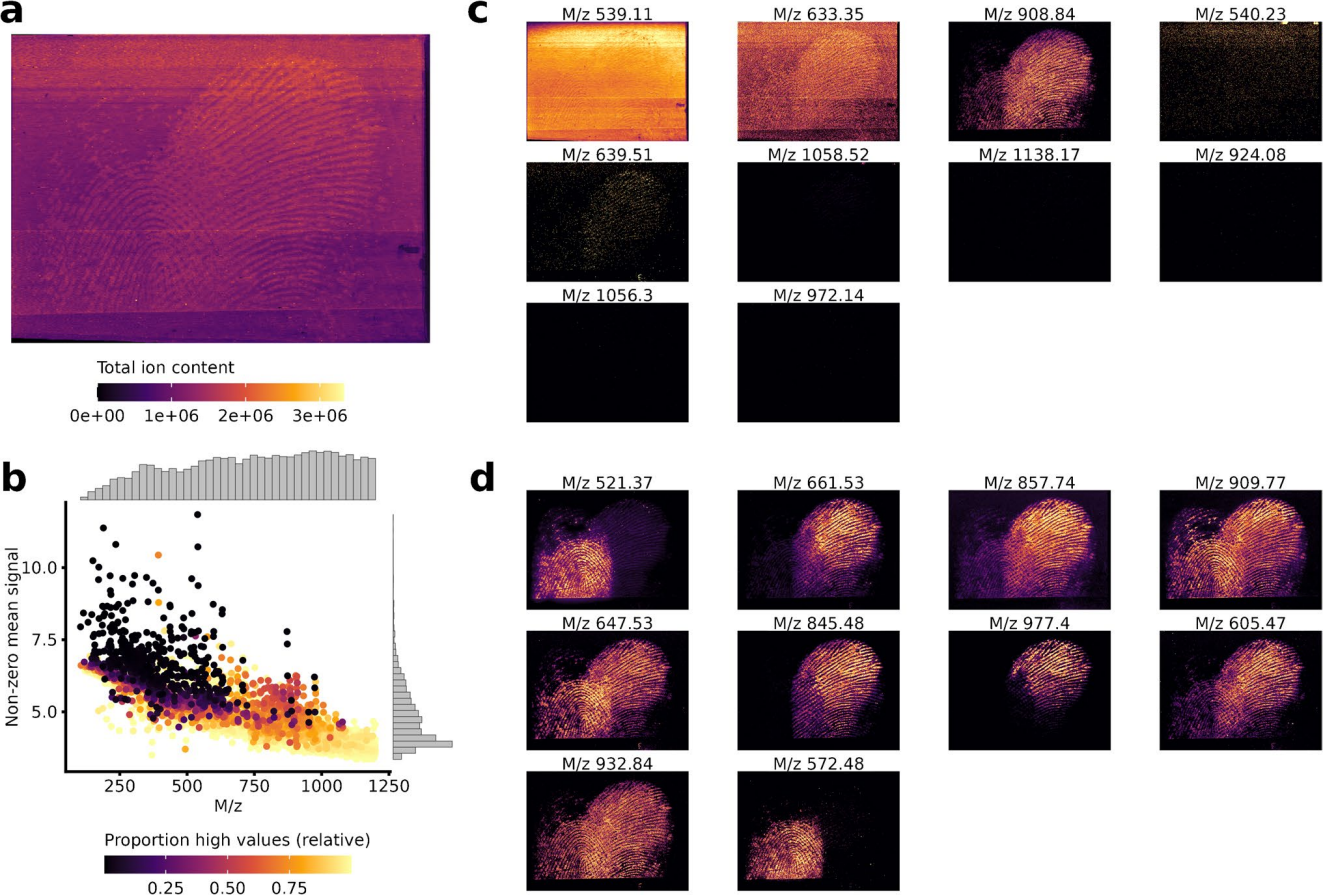
DESI-MSI data overview (Slide 3). (**a**) Spatial image using total ion content of the gel with two overlapping fingermarks from different donors. The left fingermark (Donor D12) and right fingermark (Donor D11) show distinct ridge orientations but substantial spatial overlap. (**b**) Distribution of m/z values and non-zero mean signal. Individual features are colored by the proportion of pixels with high relative intensity (intensity > 10% of max intensity). (**c**) Representative individual m/z channel images with decreasing overall signal. (**d**) Manually selected m/z channel images that localize to specific fingermark regions

DESI-MSI analysis of overlapping fingermarks yielded datasets containing approximately 100–200,000 pixels with intensity measurements across 5000 m/z channels. Figure 1a displays the total pixel signal image, revealing two distinct overlapping fingermarks with clearly visible ridge structures from both donors (Slide 3, the largest sample with most pixels). The left fingermark (Donor D12) shows a partial pattern with a continuous ridge flow around the center of the mark, while the right fingermark (Donor D11) displays a more complete pattern with a different ridge orientation. The spatial overlap between the two fingermarks is substantial, presenting a challenging separation problem that cannot be resolved through visual inspection of the image alone.

The distribution of m/z values, associated non-zero mean signals, and proportion of high-intensity pixels (Fig. 1b) show many potentially interesting features with a low proportion of high-intensity pixels and high non-zero mean signal indicating they are non-background signals. Additionally, most compounds > 1000 m/z seem to have limited signal (nearly all pixels have same values).

Examination of individual m/z channels (Fig. 1c, d) reveals heterogeneous spatial distributions, with some masses showing uniform intensity across the entire imaging area, others displaying fingermark-specific localization, and a large proportion of m/z channels show sparse or background-level signal. This heterogeneity in spatial distribution patterns across different molecular species provides the foundation for chemometric separation of the overlapping fingermarks.

### Comparison of PCA and NMF decomposition

**Fig. 2:**
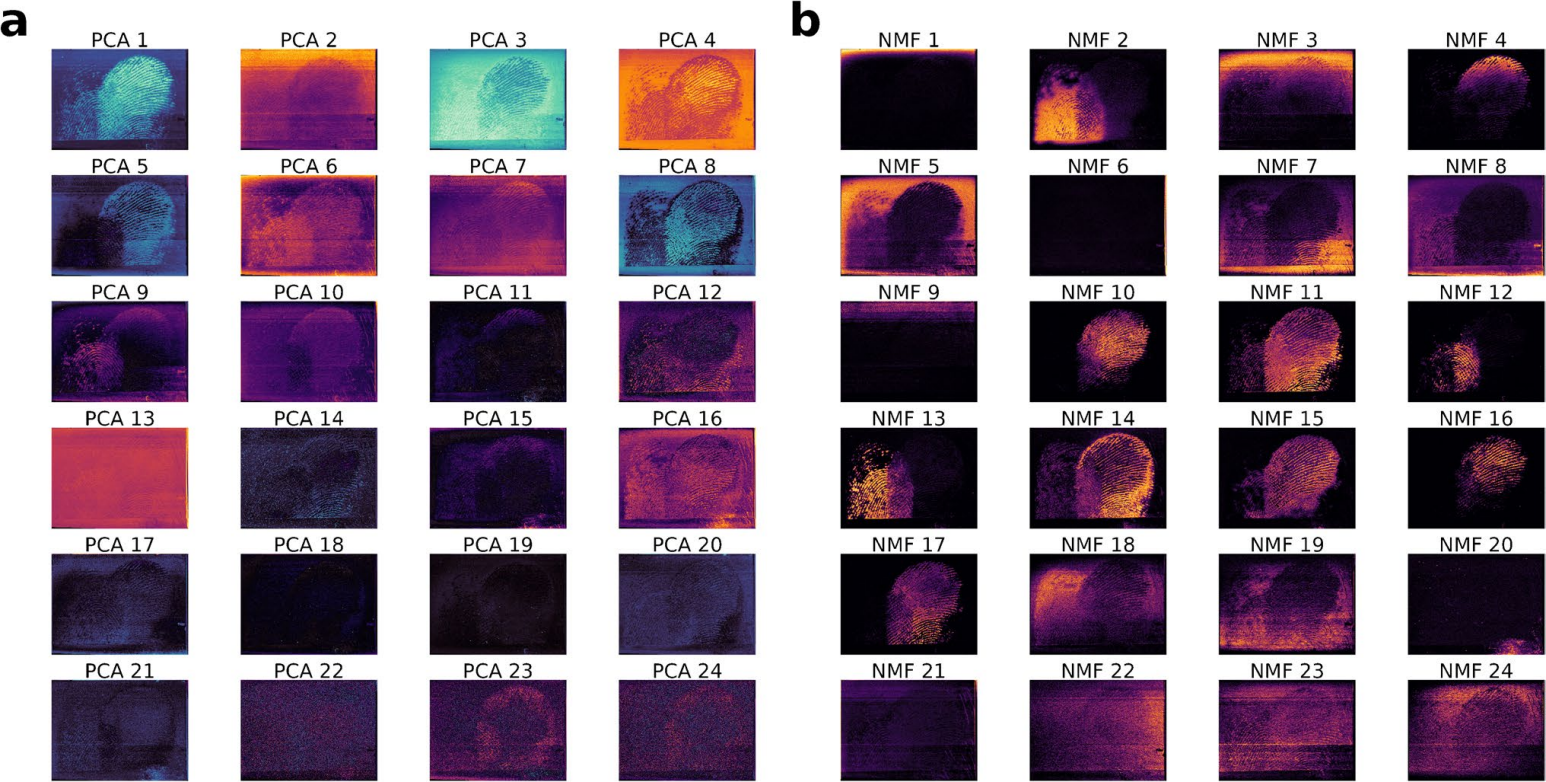
Comparison of PCA and NMF decomposition results for slide 3. (**a**) Centered PCA and (**b**) NMF spatial weight maps for the top 24 components ranked by Moran’s I spatial autocorrelation. Component weights were scaled to maximum absolute value of 1.0 for consistent color scaling. PCA components show bidirectional patterns (cyan/green = negative, yellow/orange = positive loadings), while NMF components are strictly non-negative. Note that NMF components show clearer localization to individual fingermarks compared to centered PCA

Both PCA and NMF were applied to the preprocessed data matrix, extracting 30 components each. PCA components are automatically ranked by % variance explained. NMF components were ranked by spatial coherence using Moran’s I statistic, and the top 24 components from each method are displayed in Fig. 2 for comparison.

Imaging using PCA components (Fig. 2a) naturally exhibits bidirectional spatial patterns, with positive loadings (yellow/orange) and negative loadings (cyan/green). The first principal component captures the dominant variance, primarily distinguishing fingermark regions from background, while subsequent components show increasingly complex mixtures of spatial patterns. The orthogonality constraint inherent to PCA results in components that combine multiple spatial features, making it difficult to associate individual components with distinct chemical sources. Several PCA components show fingermark ridge structure, but the patterns frequently span both donors simultaneously, reflecting correlated variance rather than donor-specific molecular signatures.

In contrast, imaging using NMF components (Fig. 2b) displays strictly non-negative spatial weights with sharper boundaries between regions. Critically, many NMF components show clear localization to individual fingermark patterns: some components are strongly expressed exclusively in the left fingermark (Donor D12), while others localize to the right fingermark (Donor D11). The parts-based decomposition produces components that correspond directly to spatially coherent molecular sources. Background regions, ridge structures, and donor-specific patterns emerge as separate components rather than being mixed within single components as observed with PCA. This pattern of qualitatively clearer localization of individual fingermark patterns was consistent across all five slides analyzed, but with qualitative differences (Figures S1–S4).

### Spatial coherence: quantitative comparison using Moran’s I

To quantitatively compare the spatial coherence of NMF and PCA components, we computed Moran’s I spatial autocorrelation statistic for all 30 components from each method. Moran’s I quantifies spatial autocorrelation by measuring whether neighboring pixels in the image have more similar values than expected by chance, ranging from −1 (dispersed) to +1 (strongly clustered). In the context of NMF and PCA, we use it to evaluate whether the spatial distributions of individual NMF and PCA components reflect biologically meaningful tissue structures rather than noise (that are expected to have low spatial structure).

**Fig. 3:**
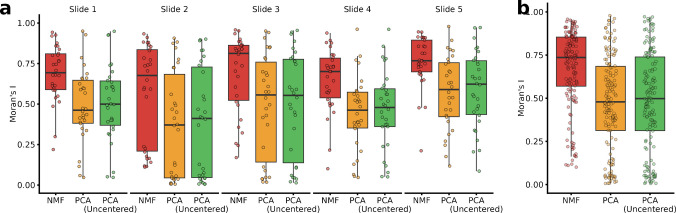
Moran’s I comparison across all five samples. (**a**) Per-sample boxplots showing Moran’s I distributions for NMF, centered and uncentered PCA. (**b**) Combined boxplot pooling all samples. NMF consistently produces significantly higher spatial coherence across all samples whereas centered and uncentered PCA produce nearly identical results

NMF exhibited a robust and significantly higher spatial coherence than both centered PCA and uncentered PCA (Fig. 3, supplementary Table 1). Moran’s I was higher for NMF across all slides which were independently acquired datasets with varying fingermark configurations. This is consistent with spatial autocorrelation analyses of other dimensionality reduction methods applied to MSI data [24]. Overall, when pooling all results (Fig. 3b), NMF had a median Moran’s I of 0.734 compared to 0.479 for centered PCA and 0.498 for uncentered PCA, with the differences confirmed by Wilcoxon rank-sum test: NMF vs centered PCA, W = 16,031, p = 2 × 10^−10^; NMF vs uncentered PCA, W = 15,507, p = 1.5 × 10^−8^. Centered and uncentered PCA produced nearly identical and non-significant spatial coherence distributions (pooled samples: W = 10,865, p = 0.61, test results for individual slides can be seen in supplementary Table 1). Per-slide tests were not corrected for multiple comparisons and conclusions regarding NMF superiority rest on the pooled analysis, which has substantially greater power. The lack of difference between centered and uncentered PCA indicates that the centering choice has negligible effect on the spatial structure of PCA components. The NMF advantage therefore arises from the non-negativity constraint and parts-based decomposition rather than from differences in data preprocessing.

### NMF component clustering reveals donor-specific signatures

**Fig. 4:**
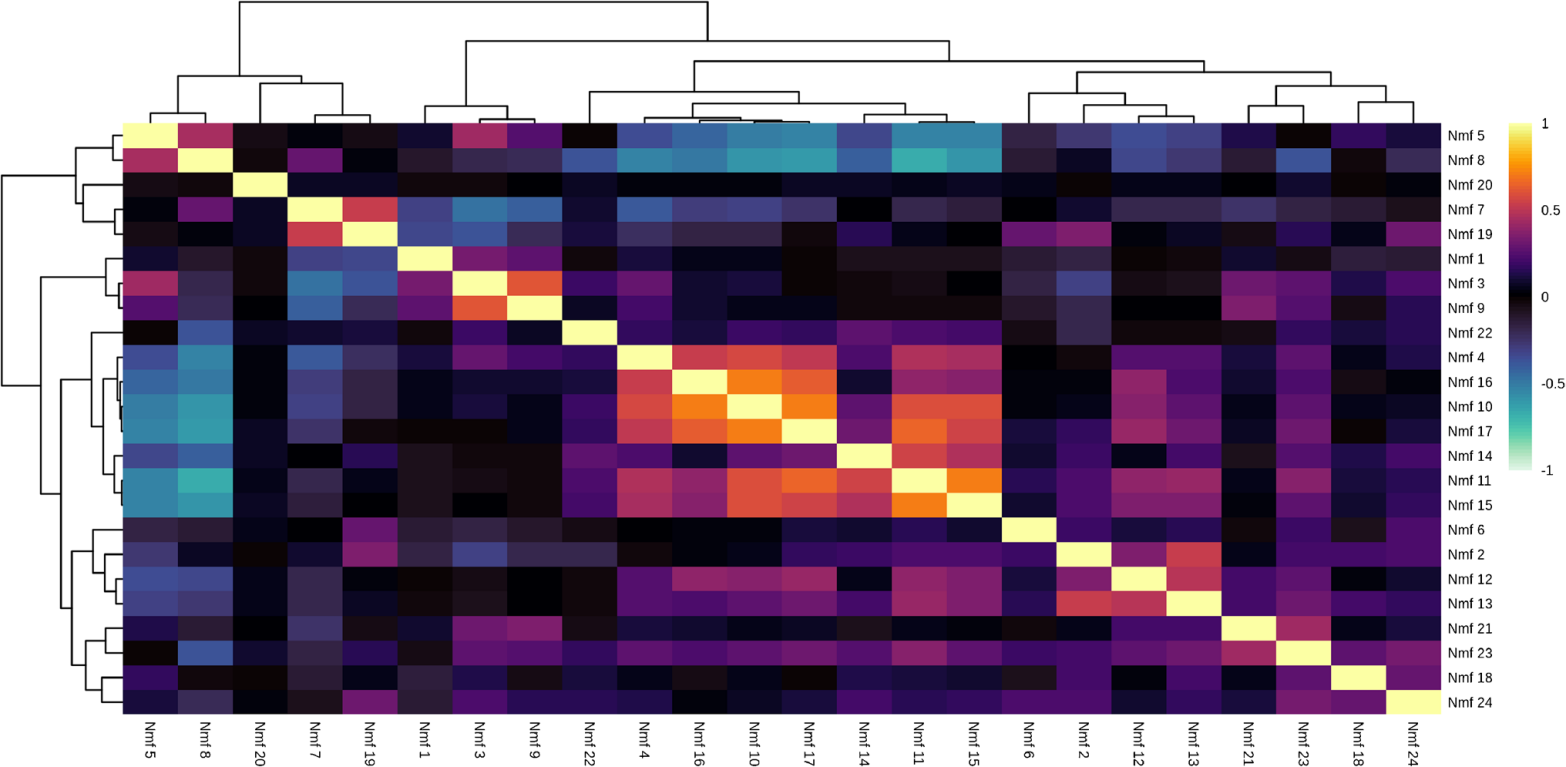
Correlation heatmap. Hierarchical clustering of NMF components using Spearman rank correlation between spatial weight vectors across all pixels. Heatmap shows individual correlation values ranging from −1 to + 1

We used Spearman rank correlation between NMF component spatial weights to cluster NMF components in order to see if visually similar spatial plots also cluster together. Hierarchical clustering reveals distinct groupings corresponding to the two fingermark donors (Fig. 4). The correlation heatmap shows two primary clusters of highly correlated components that map directly to the spatial organization of the overlapping fingermarks.

The first interesting cluster (NMF components 4, 16, 10, 17, 14, 11, 15) comprises components with spatial weight patterns localized predominantly to the right fingermark (Donor D11, see also Fig. 2b). These components exhibit strong positive correlations with each other and weak or negative correlations with components in the second interesting cluster (NMF components 2, 12, 13). This cluster captures patterns associated with the left fingermark (Donor D12, see also Fig. 2b), with similarly strong intra-cluster correlations and weak inter-cluster correlations.

Additional components form smaller clusters or remain relatively uncorrelated with the donor-specific groups, likely representing background signal, substrate contributions, or chemical features shared between both donors. Notably, this clustering emerges entirely from the unsupervised NMF decomposition without any knowledge of the number of donors.

### Extraction of donor-specific components and molecular signatures

**Fig. 5:**
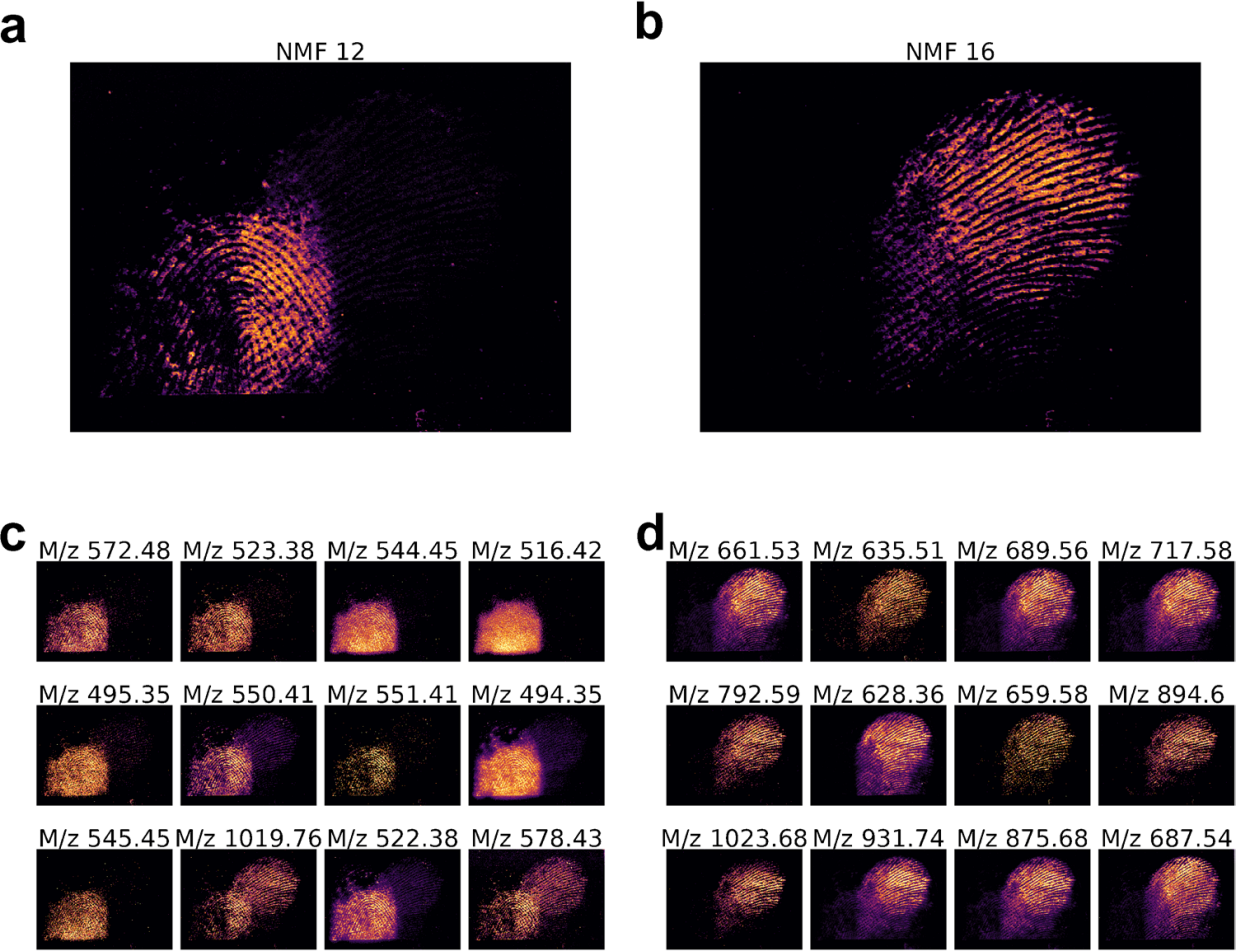
Separation of overlapping fingermarks using NMF. Spatial imaging using (**a**) NMF component 12 (left fingermark in original) and (**b**) NMF component 16 (right fingermark in original). (**c**) Top 12 m/z features with highest weights in NMF 12. (**d**) Top 12 m/z features with highest weights in NMF 16

Individual NMF components can be directly examined to identify donor-specific spatial patterns and their associated molecular signatures. Figure 5 illustrates this for two representative components from each donor cluster identified in Fig. 4.

NMF component 12 (Fig. 5a) shows spatial weights localized to the left fingermark (Donor D12), with clear ridge detail and minimal signal in the Donor D11 region. The top 12 contributing m/z features for this component (Fig. 5c) are concentrated in the 494–578 m/z range, defining the molecular signature underlying this spatial pattern. NMF component 16 (Fig. 5b) shows the corresponding pattern for the right fingermark (Donor D11), with a distinct set of high-weight m/z features (Fig. 5d) spanning a higher and broader mass range (628–1024 m/z). The two components show complementary spatial localization and largely non-overlapping molecular signatures, consistent with distinct chemical profiles from different donors.

### Molecular signatures of NMF components

The molecular basis of each NMF component is captured in the signature matrix (H), where rows represent components and columns correspond to m/z values (Figure S6a). The annotation tracks indicate mean intensity and m/z value for each feature, providing context for interpreting signature coefficients. Hierarchical clustering of normalized signatures reveals groups of components with similar molecular profiles, partially mirroring the spatial clustering observed in Fig. 4.

Components within the same spatial cluster tend to share molecular features while maintaining distinct signature profiles, suggesting that the desired number of NMF components was higher than necessary, resulting in the multiple related but distinguishable molecular signatures. NMF 9, 19, and 12 show strong coefficients concentrated in a subset of m/z features in the lower mass range, while components NMF 10 and 16 display broader signatures spanning the full mass range.

The relationship between signature coefficient and m/z value varies systematically across components (Figure S6b). Each panel displays the normalized coefficient for all m/z features within a single NMF component, colored by mean intensity. Most components show a concentration of high-coefficient features in the 200–600 m/z range, consistent with fatty acids, glycerolipids, and other small molecules characteristic of fingermark residue. Some components show more restricted signatures with high coefficients for relatively few m/z values, suggesting capture of specific molecular species rather than broad chemical classes. NMF 2, 19 and 20 have few high-coefficient features, and comparison with Fig. 2b indicates these are background or artefact signals.

Overall, the results demonstrate the practical utility of NMF for forensic applications: starting from a single unsupervised decomposition, individual fingermarks can be visualized, and donor-specific molecular features can easily be identified without prior knowledge of donor number, chemistry or manual selection of donor-specific pixels. Putative identification of specific compounds from the m/z values in each signature is beyond the scope of this work but would follow the same established workflows used for any MSI analysis, regardless of whether PCA or NMF was used for the initial decomposition.

### Simulated four-fingermark overlay

The simulated four-fingermark dataset contained approximately 5500 unique m/z channels after 0.1 Da binning. The total ion count map of the merged image (Figure S7a) shows the spatial arrangement of the four fingermark regions from donors D5, D6, D11, and D12. NMF decomposition with K = 40 components produced spatially coherent results comparable to the individual two-fingermark analyses. The distribution of Moran’s I values for the overlay dataset was broadly similar to those of the two source sessions (Figure S7b), indicating that the spatial quality of the decomposition was maintained in the more complex four-donor scenario. The top-ranked NMF components (Figure S7c) clearly resolved spatially distinct fingermark regions, with individual components localizing to fingermarks from specific donors.

## Discussion

### Successful separation of overlapping fingermarks

The primary finding of this study is that NMF successfully separates overlapping fingermarks from DESI-MSI data in a completely unsupervised manner. The clustering of NMF components into donor-specific groups emerged naturally from the decomposition without any prior information about the number of donors or their chemical characteristics. This unsupervised separation capability has significant practical implications for forensic investigations, where the number and identity of individuals who have touched a surface is typically unknown.

The quality of separation achieved is notable given the substantial spatial overlap between the two fingermarks in our test case. Individual NMF components showed clear localization to single donors with minimal signal in the opposing fingermark region, and the molecular signatures of donor-specific components were largely non-overlapping. This suggests that the chemical differences between individuals’ fingermark residues are sufficiently distinctive to enable separation even when physical overlap is extensive. Importantly, NMF produced spatially coherent donor-specific components across all five independently acquired samples (Figures S1–S4), demonstrating that the method generalizes beyond a single dataset and is robust to variation in fingermark configuration and acquisition conditions.

### Advantages of NMF over PCA for fingermark analysis

The comparison between NMF and PCA reveals clear quantitative and qualitative advantages of NMF for forensic fingermark applications. NMF components exhibited significantly higher spatial coherence as measured by Moran’s I (median 0.813 vs 0.556 for centered PCA in Slide 3, the main sample; Fig. 3). The same result was consistent across all samples (pooled median 0.734 vs. 0.479, pooled Wilcoxon test, p = 2 × 10^−10^; Fig. 3). Importantly, centered and uncentered PCA produced nearly identical spatial coherence distributions (pooled p = 0.61; Figure S5), demonstrating that the NMF advantage arises from the decomposition method itself rather than preprocessing choices. While both methods identified spatial structure in the data, NMF components showed clear localization to individual donors, whereas PCA components frequently mixed signals from both fingermarks. This difference arises from the fundamental mathematical properties of each method: PCA’s orthogonality constraint forces each component to capture variance that is uncorrelated with previous components, which does not align with the physical reality of overlapping fingermark deposits where multiple chemical sources contribute additively to each pixel.

The non-negativity constraint of NMF provides an additional advantage for interpretation. Each NMF component’s molecular signature represents compounds that are positively associated with that spatial pattern, facilitating identification of donor-specific markers or contaminants of forensic interest. In contrast, PCA loadings by design include both positive and negative values, representing compounds that are elevated or depleted relative to the mean, which is less intuitive for characterizing chemical sources.

### Methodological considerations

Several methodological choices influenced our results. We used 30 components for both NMF and PCA, then ranked NMF components by Moran’s I spatial autocorrelation statistic for display. This approach ensures that the most spatially coherent patterns appear first, facilitating visual comparison. To facilitate selection of K, the total Moran’s I across components was inspected at K = 10, 20, and 30. The incremental gain in median Moran’s I diminished substantially between K = 20 and K = 30, and K = 30 was judged sufficient to resolve all visually distinct spatial patterns in all five slides. The component correlation heatmap (Fig. 4) likewise showed no evidence of major unfactored spatial structure at K = 30. We acknowledge that a formal quantitative criterion such as cophenetic correlation could be applied in future work to make this selection more objective.

We did not apply any L1 regularization (Lasso) in the current analysis. While regularization promotes sparsity in both weight and signature matrices, the non-regularized decomposition performed well for fingermark separation in our datasets. Future work could explore the effects of different regularization parameters on separation quality and interpretability.

Log-transformation of intensity values prior to decomposition reduces the dynamic range and stabilizes variance, which improves the performance of both PCA and NMF. Alternative preprocessing approaches, such as total ion count normalization or probabilistic quotient normalization, may be appropriate for different experimental contexts.

### Practical considerations for forensic application

In practice, a forensic examiner would apply the NMF workflow as follows. After DESI-MSI acquisition and preprocessing, NMF decomposition with K = 20–30 components is run as a single unsupervised step and requires up to 25 min as currently implemented. NMF results are outputted as a single image with each NMF result as a layer for easy combination or removal of NMF layers. After identification of donor-specific NMF components candidate m/z are easily extracted for downstream identification, for example by tandem mass spectrometry. The entire computational pipeline, from raw data to donor-separated images, will run in under one hour on a standard workstation (64 GB RAM) and requires no manual parameter tuning beyond the choice of K (where a default of 30 works fine).

Although this study examines the two-donor case, the NMF framework naturally extends to scenarios with more than two contributors. Because NMF models the data as an additive combination of K components, increasing the number of donors would simply increase the number of components required to capture each donor’s spatial pattern. The visual results combined with the correlation-based clustering approach demonstrated here (Fig. 4) would then reveal multiple donor-specific clusters rather than two. The results from the overlay dataset support the scalability of the NMF approach to more than two overlapping donors, but validation under authentic multi-donor acquisition conditions remains an important next step (relevant in the context of, e.g., door handles). Overall, the additive nature of NMF provides a principled basis for multi-donor separation that PCA’s orthogonality constraint does not offer.

### Limitations and future directions

This study demonstrates the feasibility of NMF-based fingermark separation across five independently acquired samples from a controlled experimental design with six known donors. Real forensic cases may present additional challenges including partial fingermarks, degraded samples, surface contamination, and unknown numbers of contributors. Further validation across diverse sample types and conditions is needed to evaluate these effects.

The current approach does not incorporate temporal information about deposition order, which may be forensically relevant. Recent work has explored age estimation of fingermark deposits using chemical markers [25], and integration of such approaches with NMF-based separation could provide additional investigative information.

Future methodological developments could explore spatially-aware variants of NMF that explicitly model spatial autocorrelation [14, 26], potentially improving boundary definition between overlapping deposits. Filtering of features based on a database of unwanted background features (gelatine, powder) would decrease computation time and possibly improve separation. Deep learning approaches for source separation (e.g., non-linear variational auto encoders) may also offer advantages for this application and will be explored.

## Conclusion

This study demonstrates that non-negative matrix factorization can successfully separate overlapping fingermarks from DESI-MSI data in a completely unsupervised manner. The method correctly identified distinct chemical signatures associated with each donor, with individual NMF components showing clear spatial localization to single donors and minimal signal from the opposing fingermark region. Compared to PCA, NMF offers three key advantages for forensic fingermark analysis. First, the non-negativity constraint produces components that can be directly interpreted as additive chemical sources, aligning with the physical reality of fingermark deposition. Second, the parts-based decomposition naturally separates donor-specific patterns rather than mixing them within single components. Third, the molecular signatures associated with each component can be examined for compounds of forensic interest, potentially revealing information about donor characteristics or activities.

For forensic practitioners, we recommend NMF as the primary analytical method for DESI-MSI fingermark data where separation of overlapping deposits is required. PCA remains valuable for rapid exploratory analysis and quality assessment but is less suitable for detailed source separation. The completely unsupervised nature of the NMF approach means it can be applied without prior knowledge of donor number or chemical characteristics, making it well-suited for forensic investigations where such information is typically unavailable.

This work represents an initial demonstration using controlled samples, with consistent results across five independently acquired datasets, suggesting that the approach is likely to generalize across diverse forensic scenarios and is easy to adopt for case work. The results establish NMF-based chemical signature decomposition as a promising approach for addressing the longstanding challenge of overlapping fingermarks in forensic science.

## Supplementary Information

Below is the link to the electronic supplementary material.

Supplementary file1 (DOCX 5.68 MB)

## Data Availability

All data and code are available at https://zenodo.org/records/19659667.

## Declarations

### Fingermarks

Written consents were obtained from all participants including consents to publish mass data and fingermark images. Fingermarks and mass data were anonymized.
