# Supplementary material for: Automated separation of overlapping fingermarks by non-negative matrix factorization of DESI mass spectrometry imaging data
Source: Anal Bioanal Chem. 2026 May 28;418(14):4321–32. doi: 10.1007/s00216-026-06574-3 (PMC13375766; doi:10.1007/s00216-026-06574-3)
Supplement: Supplementary file 1 — Supplementary file1 (DOCX 5.68 MB) [file 216_2026_6574_MOESM1_ESM.docx]

# Supplementary information

**Supplementary Table 1**. Moran’s I comparison across methods and samples. Median Moran’s I values, test statistic (W) and associated p-values (p) from Wilcoxon rank-sum tests comparing NMF and PCA within slides.

| Slide ID | Median NMF | Median cPCA^a^ | Median ucPCA^b^ | NMF vs cPCA | | NMF vs ucPCA | | cPCA vs ucPCA | |
| --- | --- | --- | --- | --- | --- | --- | --- | --- | --- |
|  |  |  |  | **W** | **p** | **W** | **p** | **W** | **p** |
| 1 | 0.694 | 0.462 | 0.499 | 686 | 0.0004 | 661 | 0.0015 | 432 | 0.7973 |
| 2 | 0.676 | 0.371 | 0.411 | 628 | 0.0080 | 616 | 0.0136 | 436 | 0.8430 |
| 3 | 0.813 | 0.556 | 0.553 | 616 | 0.0136 | 610 | 0.0176 | 444 | 0.9357 |
| 4 | 0.701 | 0.462 | 0.479 | 690 | 0.0003 | 663 | 0.0014 | 429 | 0.7635 |
| 5 | 0.767 | 0.590 | 0.623 | 660 | 0.0016 | 632 | 0.0067 | 429 | 0.7635 |

^a^ cPCA = Centered PCA. ^b^ ucPCA = Uncentered PCA.


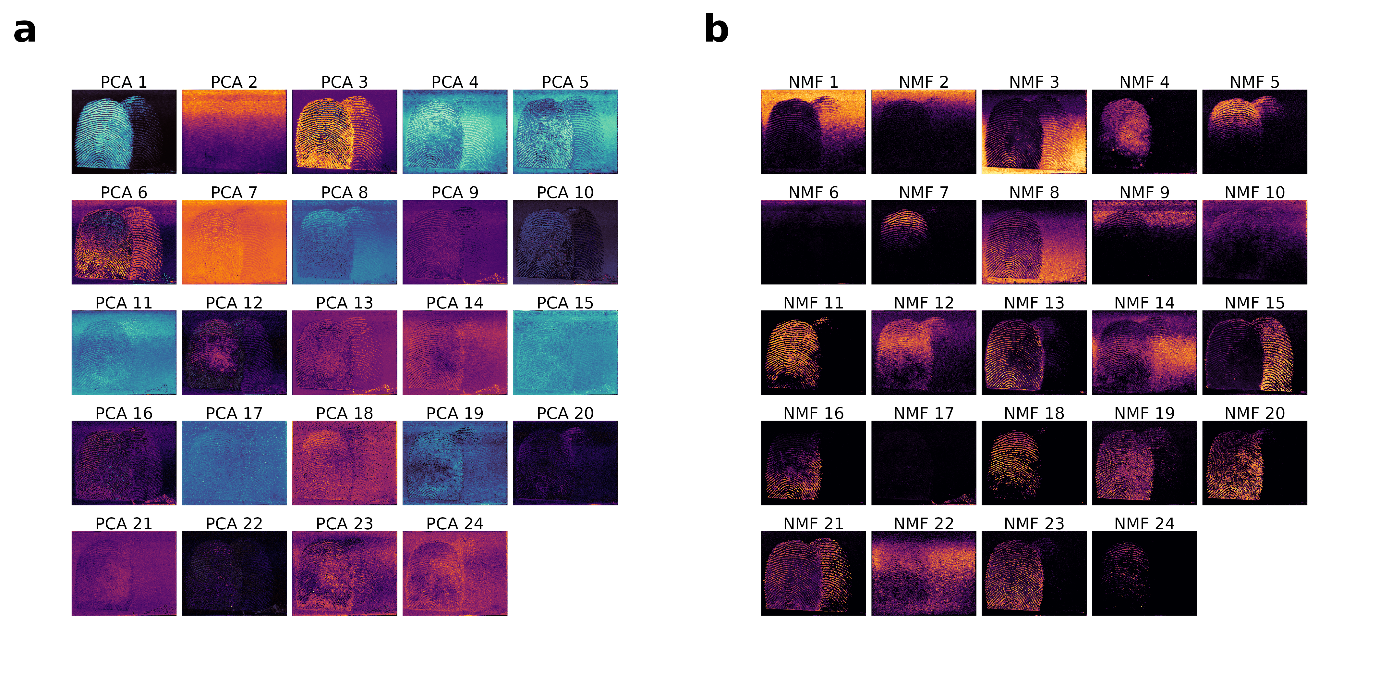


Figure S1. Centered PCA and NMF spatial component maps (top 24, ranked by Moran’s I) for slide 1.


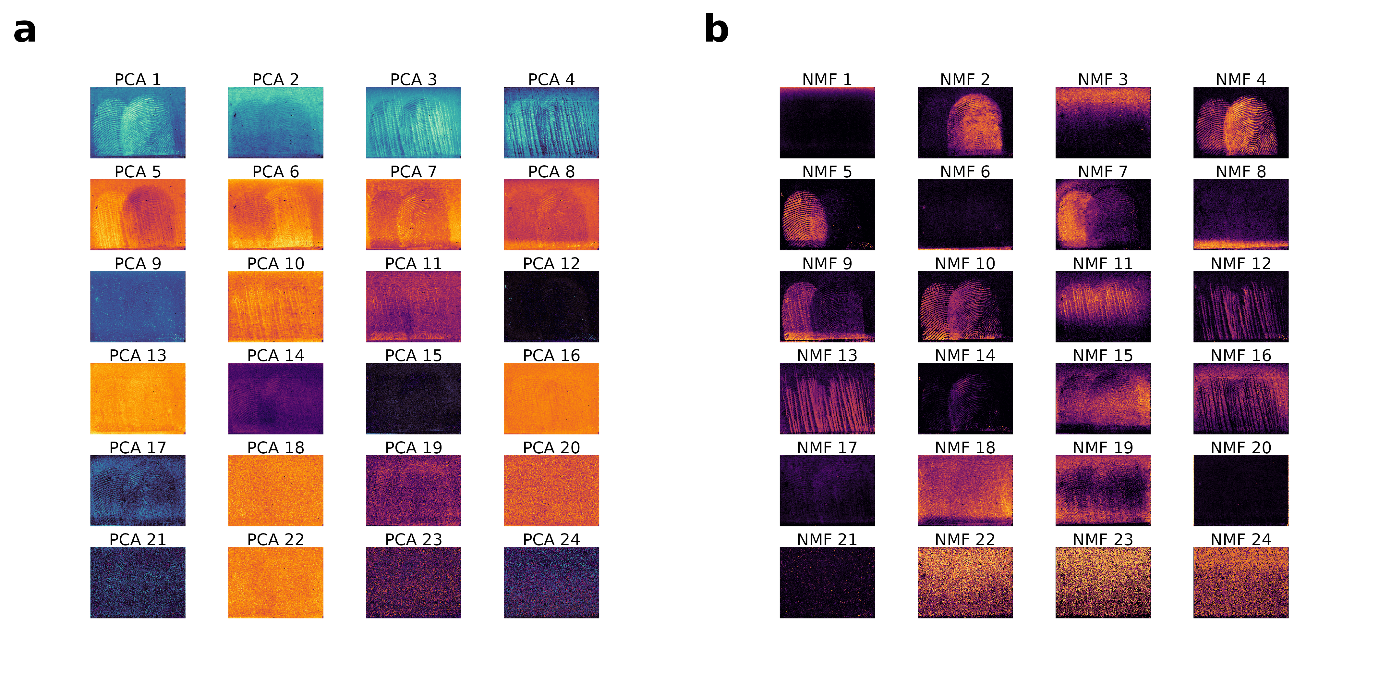


Figure S2. Centered PCA and NMF spatial component maps (top 24, ranked by Moran’s I) for slide 2.


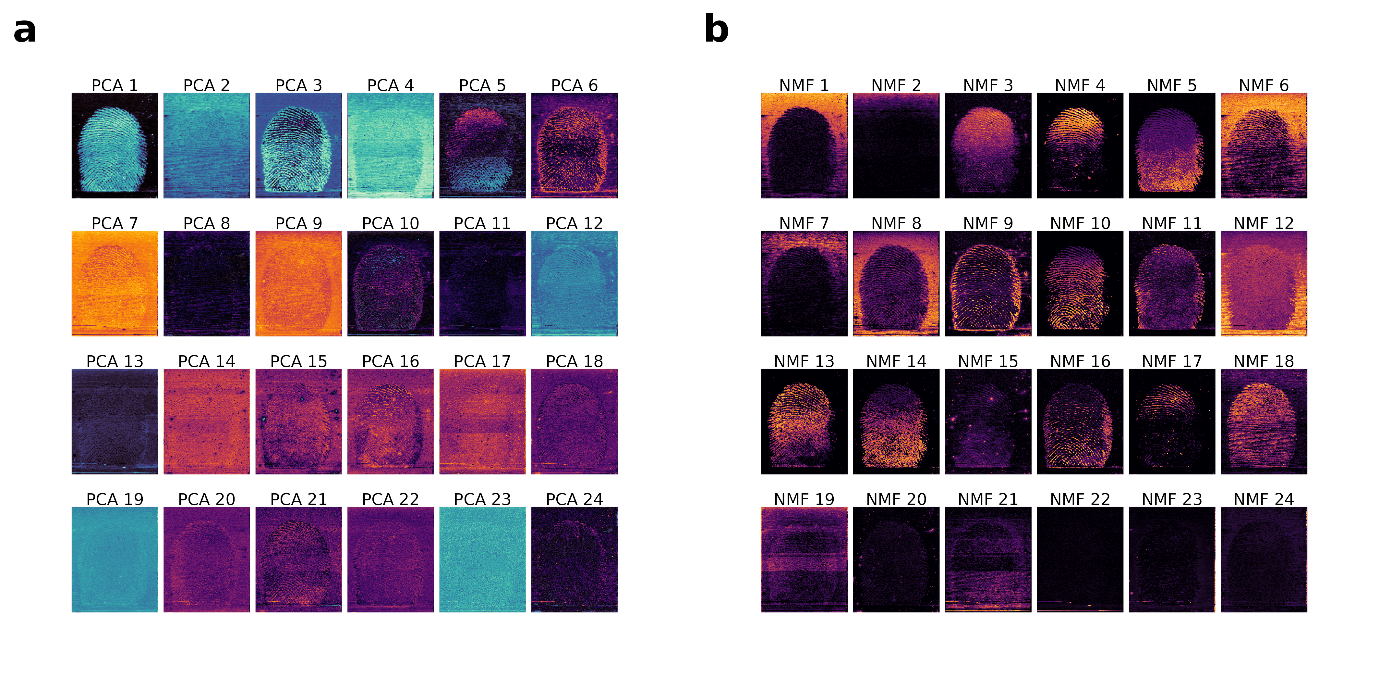


Figure S3. Centered PCA and NMF spatial component maps (top 24, ranked by Moran’s I) for slide 4.


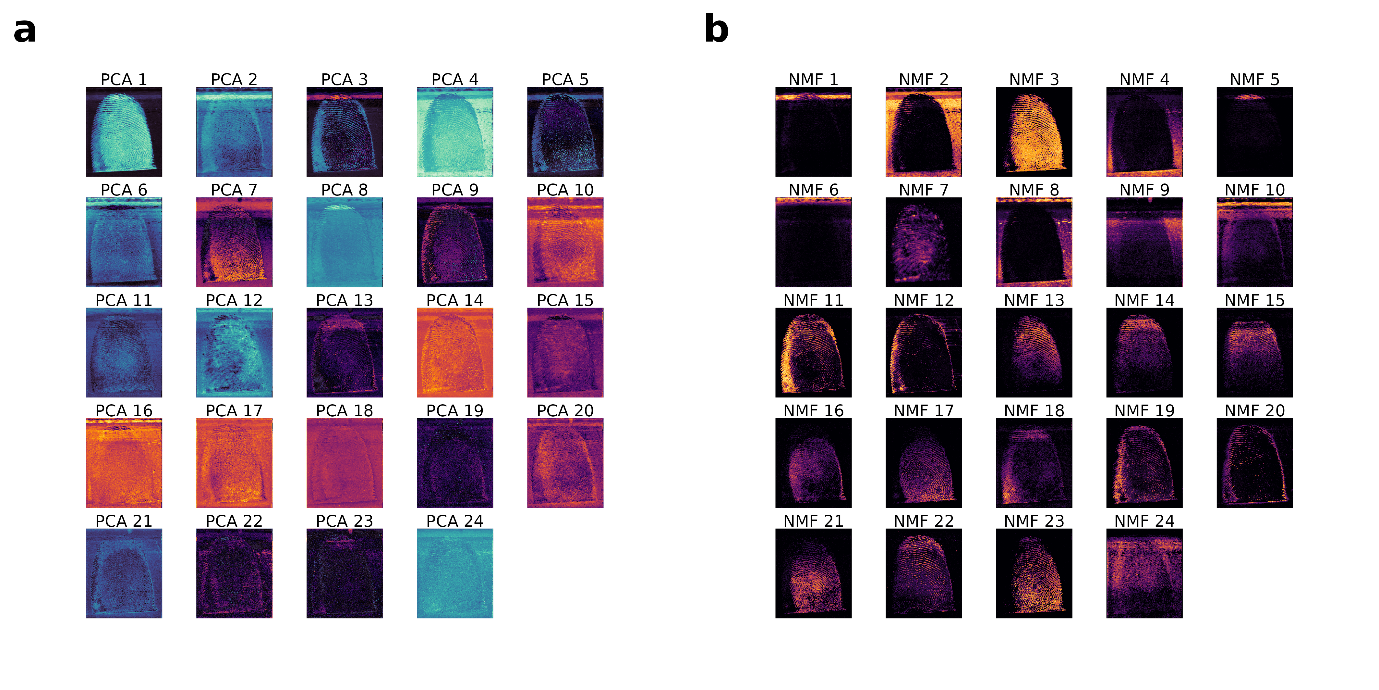


Figure S4. Centered PCA and NMF spatial component maps (top 24, ranked by Moran’s I) for slide 5.


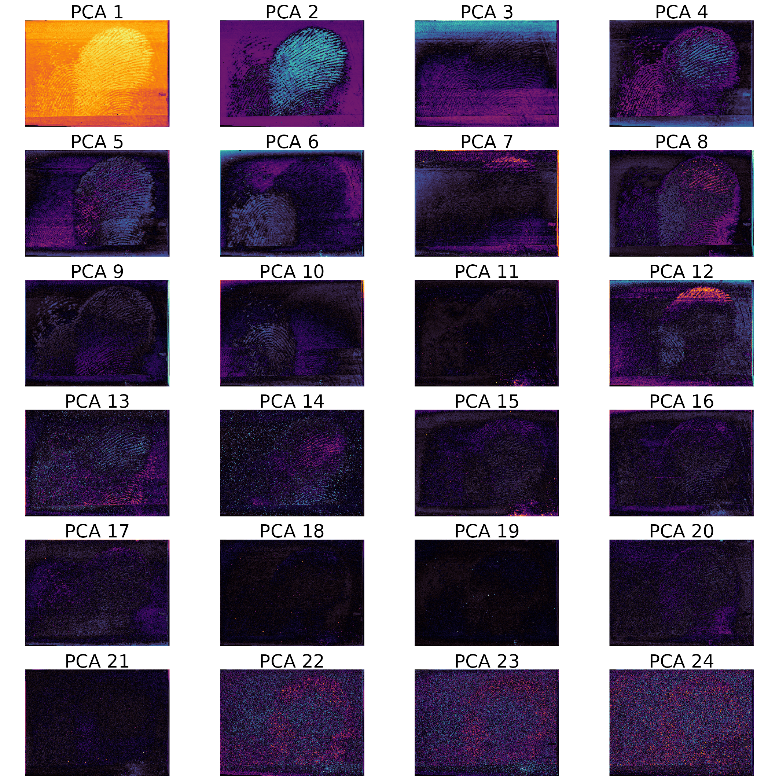


Figure S5. Uncentered PCA spatial components (top 24) for the main sample (Slide 3). Components are ordered by Moran’s I. Centered and uncentered PCA produced very similar spatial patterns and nearly identical Moran’s I distributions (Wilcoxon p = 0.94).


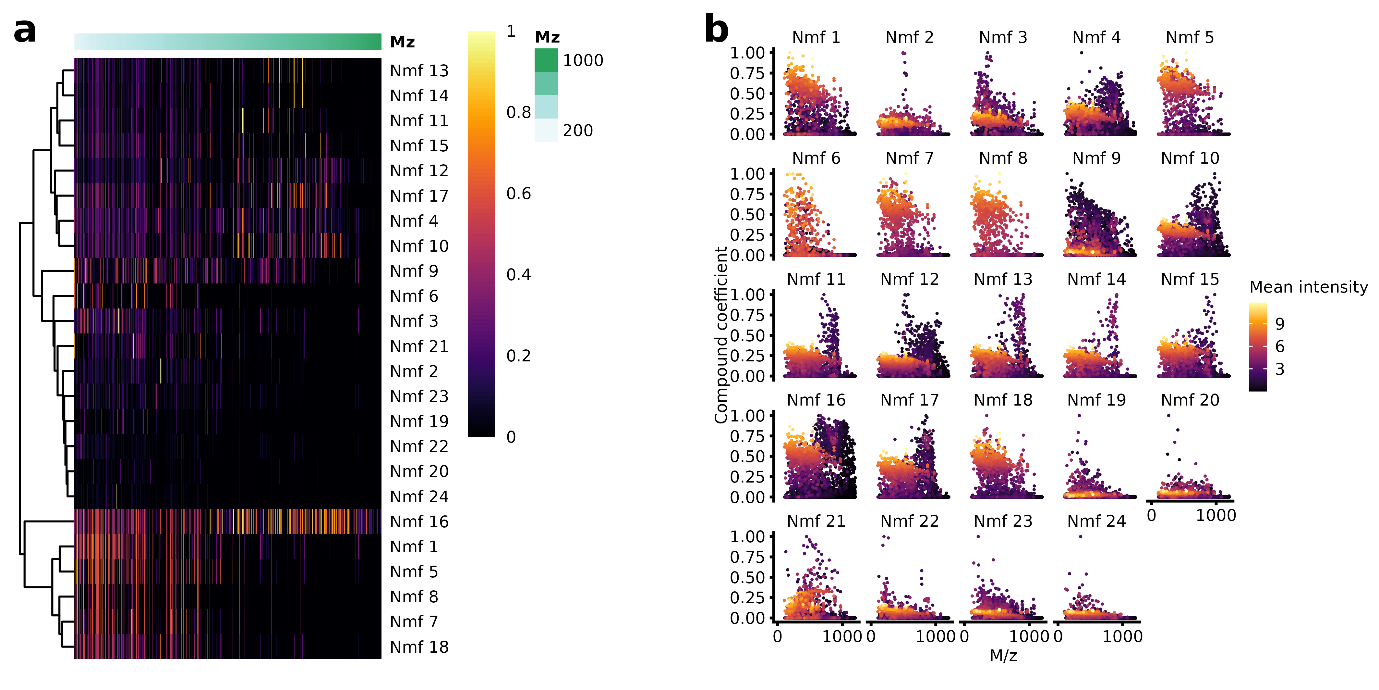


Figure S6. Molecular signatures of top 24 NMF components. (a) Heatmap showing normalized signature coefficients for each NMF component (rows) across all m/z features (columns). The column annotation indicates the m/z value for each feature. Hierarchical clustering groups components with similar molecular profiles. (b) Scatter plots showing the relationship between signature coefficient and m/z value for each of the NMF components, colored by mean intensity across the dataset.


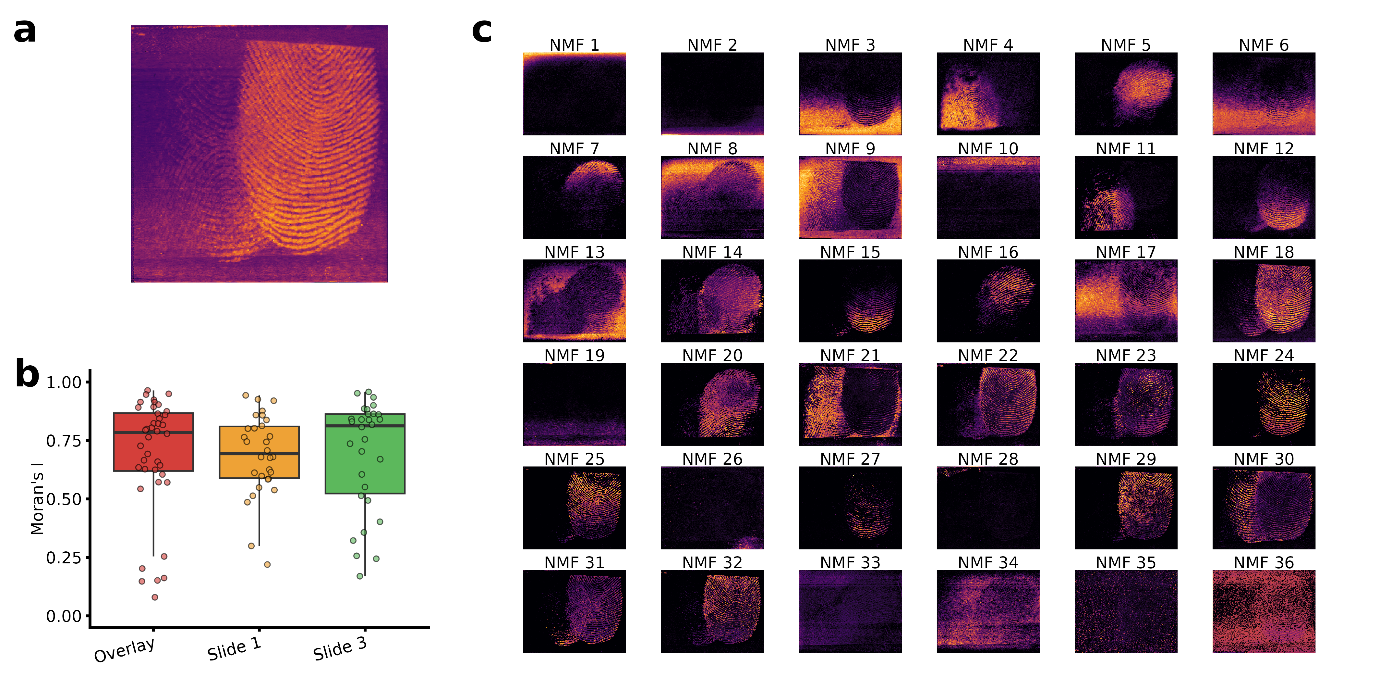


Figure S7. NMF analysis of a simulated four-fingermark dataset. Two existing acquisition sessions (slides 230215 and 231108), each containing two overlapping fingermarks, were merged after 0.1 Da m/z binning and global intensity normalization to produce a pseudo-overlay dataset. (a) Total ion count (TIC) map of the merged image, showing the spatial arrangement of the four fingermark regions. (b) Moran’s I distributions for the overlay dataset and the two source sessions; box plots show the median, interquartile range, and individual component values. (c) Spatial maps of the top 36 NMF components ranked by Moran’s I, illustrating spatially coherent and distinct patterns across the four-fingermark dataset.
